# Refractory Kawasaki Disease Presenting With Erythema at Bacille Calmette-Guérin Inoculation Site: A Paediatric Case Report

**DOI:** 10.7759/cureus.10928

**Published:** 2020-10-13

**Authors:** Vijayakumary Thadchanamoorthy, Kavinda Dayasiri

**Affiliations:** 1 Clinical Sciences, Faculty of Health-Care Sciences, Eastern University, Batticaloa, LKA; 2 Internal Medicine: Paediatrics, Base Hospital, Mahaoya, LKA

**Keywords:** refractory, bcg site erythema.

## Abstract

Kawasaki disease (KD) is an autoimmune disease that generally affects children under the age of five years. It has a variety of clinical manifestations which may be either specific or nonspecific. Intravenous immunoglobulin and aspirin are the mainstays of treatment. There are unusual circumstances where patients are resistant to conventional treatment. We report a one-year-old girl who presented with a 12-day history of fever in association with erythema at the site of Bacille Calmette-Guérin (BCG) scar. She did not respond successfully to conventional treatment although she was diagnosed to have Kawasaki disease. Eventually, she responded to intravenous methylprednisolone and was diagnosed as having refractory Kawasaki disease.

## Introduction

Kawasaki disease (KD) is an acute, self-limiting autoimmune vasculitis that affects small- and medium-sized arteries and also is now the most common cause of acquired heart disease in children under five years in the developed world [1,2]. Tomisaku Kawasaki first defined a case of KD in January 1961 and the report was published in 1967. A significant physical sign that is not counted in classical clinical criteria for KD is the reaction at Bacille Calmette-Guérin (BCG) inoculation site. Erythema at BCG inoculation site exists in approximately 30%-50% of KD patients [3]. Approximately 25% of children with KD, who do not receive treatment early in the course of the disease, will develop coronary artery aneurysms (CAA) [4]. The cornerstone of traditional therapy is intravenous immunoglobulin (IVIG) and aspirin therapy. There is a growing incidence of patients who do not respond to combined IVIG therapy and aspirin. It is identified as IVIG-resistant or refractory KD where recrudescent or persistent fever is present at 24-48 hours following the first IVIG infusion. The refractory disease accounts for 10%-20% of patients with KD [5]. We report a child who did not respond to two doses of IVIG and aspirin and in whom fever responded only following the administration of intravenous methylprednisolone.

## Case presentation

A one-year-old previously healthy child presented to the general practitioner (GP) with a history of fever, diarrhea, and cold for five days, and erythema at the BCG inoculation site for one-day duration. She had been initially treated as bacillary dysentery with oral cotrimoxazole. While on treatment, she developed strawberry tongue and swelling of hands and feet along with the appearance of conjunctivitis and persistent high fever. The child was admitted only on day 12 of illness with suspicion of Steven Johnson syndrome secondary to cotrimoxazole. Further, the child had poor feeding and watery discharge from her right ear. She had age-appropriate immunization and there was no history of allergy to drugs, food, and environmental allergens.

**Figure 1:**
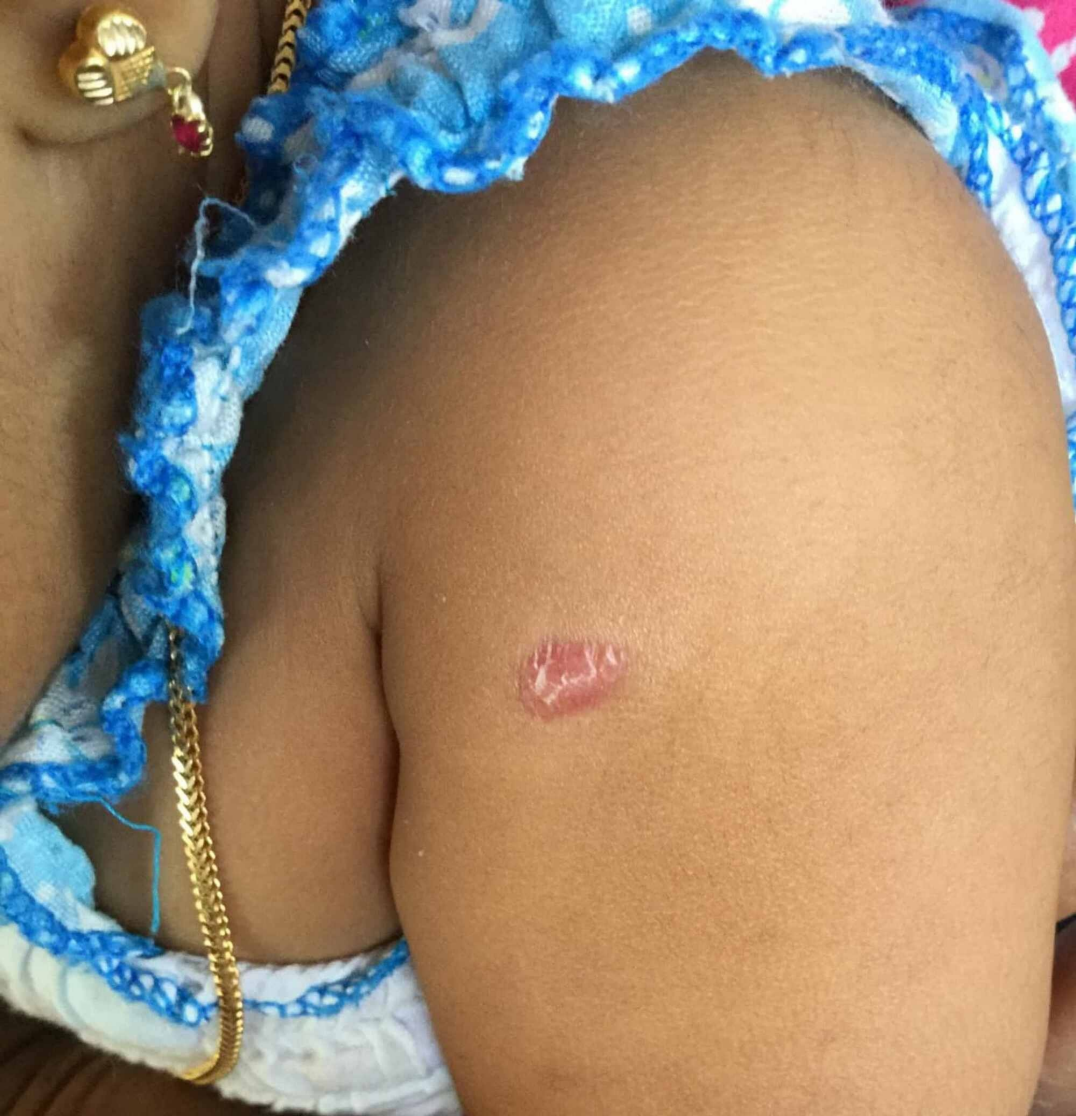
Erythema at the site of BCG scar BCG, Bacille Calmette-Guérin

Physical examination revealed that she was ill, febrile (above 102 F), irritable, dehydrated, and had bilateral conjunctivitis without discharge. There was cervical lymphadenopathy measuring 2 cm in size. Feet and hands were swollen. Lips were cracked with beefy red tongue and a well-defined erythematous reaction was evident at the BCG inoculation site (Figure 1). Other systems examination was normal apart from having mild right hypochondrial tenderness.

Investigations revealed a high white blood count (18x10^3^ /cumm (normal range - 6x10^3^ -17x10^3^/cumm) with neutrophil 75%), low haemoglobin (8.9 g/dl - normal range 11.3 g/dl - 14.1 g/dl), normal platelets (350x10^3^/cumm), high C-reactive protein (CRP-148 mg/dl - normal range - less than 5 mg/dl ), and high ESR (120 mm/hour). Blood picture showed evidence of either infection or inflammation with the left shift of neutrophils. Urinalysis showed microscopic haematuria (5-8/HPF) and pyuria (10-14/HPF). The cerebrospinal fluid analysis revealed normal findings including cultures apart from showing slightly high protein (60mg/dL). Serum sodium was 134meq/l, and potassium was 4.2meq/l. Serum total protein was 60mg/dl, and albumin was 35mg/dl. Aspartate transaminase (154 IU/l) and alanine transaminase (104 IU/L) were elevated. Alkaline phosphatase was normal (300 IU/L). Renal functions had been normal except for elevated blood urea (70mgdl). Throat swab showed no organisms, and blood and urine cultures were sterile. Rheumatoid factor was negative. Antistreptolysin O titer (ASOT) was less than 200 IU/L. Antibody to leptospira, mycoplasma, Epstein Barr virus, and scrub typhus were negative. Chest X-ray was normal. Ultrasound abdomen showed hydrops of the gall bladder, and otherwise findings were normal. Echocardiogram and electrocardiogram revealed normal findings with no evidence of coronary aneurysms. The ophthalmological examination had been normal.

According to the American Heart Association (AHA) guidelines, she had fulfilled the criteria of typical or complete KD with non-specific signs especially erythema at BCG scar and irritability. She was treated with intravenous immunoglobulin (IVIG) at the dose of 2g/kg and the anti-inflammatory dose of aspirin (100mg/kg). As the child did not show any improvement, the second dose of IVIG was also given 48 hours after the first dose. Despite treatment, she remained ill and irritable and high fever persisted. The repeat investigations showed further rising white blood cells (total- 25x10^3^ /cumm, neutrophils-98%), CRP (248 mg/dl), ESR (140 mm/hour) and platelets (746x10^3 ^/cumm). A repeat echocardiogram at this stage showed coronary artery abnormalities including dilatation of the left coronary artery (2.8mm, > 95th centile). The diagnosis of refractory KD was made based on clinical, echocardiographic, and laboratory findings after initial treatment with IVIG, and she was commenced high dose intravenous methylprednisolone (30mg/kg) which was subsequently continued for five days. Fever subsided after the third dose of intravenous methylprednisolone. High dose aspirin was continued until inflammatory markers became normal. Subsequently, the dose was changed to an anti-platelet dose of aspirin (5mg/kg) and continued up to six weeks. Repeat echocardiogram revealed persistent dilatation of the left coronary artery measuring the same diameter noted at the initial diagnosis of coronary disease. She was kept on long term clinic follow up with low dose aspirin and echocardiographic monitoring. After one year, the repeat echocardiogram showed a normal left coronary artery. Currently, she is being followed at both paediatric and cardiology clinics.

## Discussion

Dr. Tomisaku Kawasaki reported the first patient with KD in 1961 and published 50 patients with similar clinical features in 1967. It is the most common acquired heart disease during childhood in most developed countries. Abnormalities of coronary arteries are well-known complications of KD and comprise up to 15%-25% of patients if definitive treatment is not administered on time [2,3,6]. However, the etiology of KD remains unclear, and studies are ongoing to explore precise pathophysiological and genetic profiles and new treatment modalities for KD [5,7].

The diagnosis of KD is based on specific clinical criteria: fever lasting at least for five days or more with at least four out of five of the following; (1) bilateral non-purulent conjunctivitis, (2) mucosal changes of the oropharynx, (3) changes in the extremities (oedema and/or erythema of the hands or feet, desquamation, beginning periungual), (4) rash (usually truncal) polymorphous but non-vesicular, and (5) cervical lymphadenopathy which is more than 1.5cm [8]. Several non-specific features have been noticed mostly in incomplete KD and they include irritability, erythema at BCG inoculation site, and hydrops of gall bladder [5]. The erythema at the site of the BCG scar has been noticed in one study with frequency more than that of lymphadenopathy and rash in countries where the national BCG immunization program is available [3]. BCG site erythema is an important sign evident in KD, however, it is not included in the diagnostic criteria [3]. This child had erythema since day four of illness, but parents attributed the erythema for a mosquito bite until this was confirmed at the physical examination in the hospital. The physical examination further confirmed a typical KD in association with BCG erythema.

KD is a vasculitic disease with a high tendency to affect coronary arteries. The immune-regulatory mechanisms are mediated through deficiency of circulating CD8+ suppressor/cytotoxic T cells, an abundance of circulating B cells spontaneously-producing immunoglobulins, and circulating, activated monocytes [8,9]. According to the AHA protocol, IVIG, and high-dose aspirin have been the first-line treatment for the management of KD [5]. Although the action of IVIG in controlling the inflammatory process is not known, it has been thought that it has some immune-modulatory effects such as cytokine production, influence on T-cell activity, and suppression of antibody synthesis [8-10]. The reported child had satisfied the criteria for typical KD and was started conventional treatment with IVIG and aspirin. However, she did not respond even after the second dose of IVIG according to treatment defined by the AHA [5]. The diagnosis of refractory KD was made at this stage.

As she had a poor response to conventional management and clinical, echocardiographic and laboratory findings suggestive of resistant KD, the authors had to exclude other possible differential diagnosis mimicking KD including drug hypersensitivity, Staphylococcal scalded skin syndrome, scarlet fever, scrub typhus, viral infection, mycoplasma infection, systemic-onset juvenile rheumatoid arthritis, Stevens-Johnson syndrome, toxic shock syndrome, measles, leptospirosis, and certain tick-borne illnesses, such as Rocky Mountain in those conditions the coronary artery aneurysm was not a feature. Finally, the diagnosis was made as immunoglobulin-resistant KD.

A study done in Japan [11] suggested some risk factors which can be used to predict treatment-resistant KD and they include (1) initial treatment at or before the fifth day of the illness, (2) recurrent episodes of KD, (3) male sex, (4) young patient age, particularly less than one-year, (5) significantly elevated CRP, (6) elevated liver enzymes, (7) platelet count of less than 300x10^3^ cumm, (8) elevated band count, (9) serum sodium less than 133, and low serum albumin [8,9,10]. Besides, a study done in South Korea showed fewer lymphocyte percentages compared to neutrophil during the sub-acute stage helped to predict IVIG-resistant KD [12]. This patient had most of the risk factors mentioned above including young age, elevated CRP, increased band forms in the blood picture, elevated liver enzymes, and low lymphocyte during the acute stage of illness. 

Persistent and refractory fever increases the risk of developing coronary artery aneurysms [9]. AHA endorses a second dose of IVIG for persistent fever despite initial intravenous immunoglobulin. High dose (2g/kg) immunoglobulin is associated with a reduced incidence of coronary artery disease compared to low dose regimen (1g/kg) [8,11,13]. The study was done in Korea further described that the risk of development of coronary abnormalities was more among patients with IVIG-resistant KD [12]. The reported child presented late on day twelve of fever and the resistant nature of the disease to conventional treatment contributed to the development of coronary abnormalities which were not evident in the initial echocardiogram.

There are several treatment alternatives suggested for refractory KD such as corticosteroid, cyclosporine A, tacrolimus, and infliximab. High dose IV methylprednisolone (30mg/kg/dose) is recommended as a first-line initial treatment for refractory KD. The action of corticosteroids has been proposed to inhibit phospholipase A2 which is needed for the production of arachidonic acid and inflammatory markers [5,13,14]. This in turn reduces the inflammatory effect of vasculitis [15]. Patients treated with IV methylprednisolone had a faster resolution of fever and all were afebrile within one day of treatment [5,8,13,14]. The timing of steroid therapy has been controversial according to various studies done on patients with KD [15,16]. One study suggested that the use of steroids in children with poor responses to the first dose of IVIG and also children with high-risk categories benefited from a combination of steroids and IVIG as an initial treatment [15,16]. Current evidence supports that corticosteroids move from unproven to effective adjunctive therapy [15] and treatment with methylprednisolone in the early stage of the resistant cases has been shown to reduce the occurrence of coronary artery aneurysms in KD [15,16]. 

## Conclusions

The diagnosis of KD has always been a clinical-based diagnosis and should be suspected in all children with initial atypical presentations. Diagnosis and treatment of refractory KD are often challenging and refractory disease is associated with a higher risk for the development of coronary abnormalities. Erythema at BCG inoculation site although not-included in diagnostic criteria should be actively looked for thus the diagnosis of KD can be suspected without delay.
